# Transcriptomics analysis identified ezrin as a potential druggable target in cervical and gastric cancer cells

**DOI:** 10.1016/j.clinsp.2024.100422

**Published:** 2024-07-06

**Authors:** Maria Fernanda Lopes Carvalho, Carolina Santana Calicchio, Bruna Oliveira de Almeida, Livia Bassani Lins de Miranda, Jean Carlos Lipreri da Silva, Keli Lima, João Agostinho Machado-Neto

**Affiliations:** a Department of Pharmacology, Instituto de Ciências Biomédicas, Universidade de São Paulo, São Paulo, SP, Brazil; b Laboratory of Medical Investigation in Pathogenesis and Targeted Therapy in Onco-Immuno-Hematology (LIM-31), Department of Internal Medicine, Hematology Division, Faculdade de Medicina, Universidade de São Paulo, São Paulo, SP, Brazil

**Keywords:** Ezrin, Transcriptomics, Cervical cancer, Gastric cancer, Pharmacology

## Abstract

**Objective:**

Cancer genomics and transcriptomics studies have provided a large volume of data that enables to test of hypotheses based on real data from cancer patients. Ezrin (encoded by the *EZR* gene) is a highly expressed protein in cancer that contributes to linking the actin cytoskeleton to the cell membrane and signal transduction pathways involved in oncogenesis and disease progression. NSC305787 is a pharmacological ezrin inhibitor with potential antineoplastic effects. In the present study, the authors prospected *EZR* mRNA levels in a pan-cancer analysis and identified potential cancers that could benefit from anti-*EZR* therapies.

**Methods:**

This study analyzed TCGA data for 32 cancer types, emphasizing cervical squamous cell carcinoma and stomach adenocarcinoma. It investigated the impact of EZR transcript levels on clinical outcomes and identified differentially expressed genes. Cell lines were treated with NSC305787, and its effects were assessed through various cellular and molecular assays.

**Results:**

*EZR* mRNA levels are highly expressed, and their expression is associated with biologically relevant molecular processes in cervical squamous carcinoma and stomach adenocarcinoma. In cellular models of cervical and gastric cancer, NSC305787 reduces cell viability and clonal growth (p < 0.05). Molecular analyses indicate that the pharmacological inhibition of *EZR* induces molecular markers of cell death and DNA damage, in addition, to promoting the expression of genes associated with apoptosis and inhibiting the expression of genes related to survival and proliferation.

**Conclusion:**

The present findings provide promising evidence that ezrin may be a molecular target in the treatment of cervical and gastric carcinoma.

## HIGHLIGHTS

• Ezrin (EZR) is involved in oncogenesis and disease progression.
• EZR is highly expressed in cervical squamous cell carcinoma and stomach adenocarcinoma.
• NSC305787, an EZR inhibitor, reduces cell viability and clonal growth in cervical and gastric cancer cells.
• EZR may be a molecular target in the treatment of cervical and gastric carcinoma.

## Introduction

In recent years, cancer genomics and transcriptomics studies have provided and publicly deposited a large volume of data and driven the development of analysis platforms for generating and testing hypotheses based on real data from cancer patients.^1^^,^^2^ Among these studies, the initiative of The Cancer Genome Atlas (TCGA) stands out. These data allow evaluation of the impact of mutations or gene expression on clinical, biological, molecular, or survival outcomes.

Ezrin (encoded by *EZR*) is a member of the ERM (ezrin, radixin, moesin) protein family, and when phosphorylated, it is essential for linking the actin cytoskeleton to the cell membrane.^3^ Ezrin binds to integral membrane proteins, adhesion molecules, multidrug resistance proteins, scaffold proteins, Rho-related proteins, tyrosine kinases, and PIP_2_.^3^ In normal tissues, *EZR* is widely expressed, but its high levels are time-specific during human development.^4^^,^^5^ This protein also contributes to signal transduction pathways involved in cancer development and progression, including PI3K/AKT/mTOR and RhoGTPase signaling.^3^ In cancer cells, EZR-mediated AKT activation modulates the BCL2 protein family, favoring survival and proliferation.^3^ Thus, there is growing evidence that EZR may serve as a proto-oncogene, and several studies have demonstrated its high expression in tumors compared to their normal histological counterpart.^3^

Given the above, *EZR* has drawn up attention as a potential pharmacological target to compose the antineoplastic arsenal. There are two synthetic compounds described as selective ezrin inhibitors (NSC668394 and NSC305787) that have been better characterized in preclinical studies, NSC305787 being considered more potent and exhibiting a better pharmacokinetic profile.^6^^,^^7^

In the present study, the authors examined *EZR* mRNA levels across various cancers through a comprehensive pan-cancer analysis. These findings highlighted cancer types exhibiting notably elevated *EZR* expression, specifically cervical and gastric cancer. Consequently, the authors posited that these particular tumor types might offer promising targets for anti-EZR therapies. To explore this hypothesis further, the authors evaluated the efficacy of an EZR inhibitor, NSC305787, in representative cellular models of these diseases.

## Material and methods

### Bioinformatics and functional genomics analysis

Data from TCGA cohorts that included 32 types of cancer, of which 21 had data from adjacent normal tissue, were obtained from the TIMER 2.0 database (http://timer.cistrome.org/). Statistical significance was calculated using the Wilcoxon test.

Gene expression and clinical data from the TCGA cervical squamous cell carcinoma (n = 294) and stomach adenocarcinoma (n = 412) studies were obtained from cBioPortal (https://www.cbioportal.org/).^8^^,^^9^ The impact of *EZR* transcript levels on clinical outcomes (Overall Survival [OS], Disease-Free Survival [DFS], and Disease-Specific Survival [DSS]) was investigated. Dichotomization was realized according to the ROC curve and its respective Area Under the Curve (AUC) and the C-index using a maximization metric provided by the R package Cutpoint.^10^ The observational study follows the STROBE statement.

All transcripts from the RNAseq of the TCGA cervical squamous cell carcinoma and stomach adenocarcinoma cohorts were pre-ranked according to their differential expression by comparing samples with high and low *EZR* expression. Normalized quantile gene expression was used for classification using the limma-voom package at Galaxy (https://usegalaxy.org/). A heatmap was constructed using ClusterVis (https://biit.cs.ut.ee/clustvis/) to represent the top 50 differentially expressed genes between high and low *EZR* expression samples. Volcano plots correlating the adjusted -log10 p-value and log2-fold-change and Spearman correlation plot were constructed in GraphPad Prism 8.0 (GraphPad Software, Inc., San Diego, CA, USA). All differentially expressed genes obtained from the Galaxy tool (fold-change > 1.5 and p < 0.05) were used for Gene Ontology (GO) analysis using ShinyGo 0.77 database (http://bioinformatics.sdstate.edu/go/), and the top twenty upregulated and downregulated GO biological processes, GO molecular processes, and GO cellular components were illustrated.

### Cell culture and reagent chemicals

A-431 (cervix squamous cell carcinoma) and HGC-27 (metastatic gastric cancer) cells were obtained from Banco de Células do Rio de Janeiro (BCRJ; Rio de Janeiro, Brazil). AGS (gastric adenocarcinoma) cells were kindly provided by Prof. Tiago Góss dos Santos (A. C. Camargo Cancer Center, São Paulo, Brazil). Cell culture conditions were performed according to the recommendations of the BCRJ and American Type Culture Collection (ATCC). NSC305787 was purchased from ChemScene (Monmouth Junction, NJ, USA) and prepared as a 10 mM stock solution in dimethyl sulfoxide (Me_2_SO_4_; DMSO).

### Cell viability assay

Cell viability was determined by a Methylthiazoletetrazolium (MTT) assay. A total of 5 × 10^3^ cells per well were plated in a 96-well plate and exposed to vehicle or increasing concentrations of NSC305787 (ranging from 0.4 to 50 µM) for 24h. Then, 10 μL MTT solution (5 mg/mL) was added, and the cells were incubated at 37°C in 5% CO_2_ for 4h. The reaction was stopped by adding 100 μL 0.1N HCl in anhydrous isopropanol. Cell viability was evaluated by measuring the absorbance at 570 nm. The IC_50_ values were calculated by performing a nonlinear regression analysis in GraphPad Prism 8 (GraphPad Software, Inc.).

### Colony formation assay

Cancer cell lines (1 × 10^3^ cells/35 mm^2^ plate) were incubated with vehicle or different concentrations of NSC305787. Colonies were detected after 10–15 days of culture by staining with 0.5% crystal violet (Sigma-Aldrich, St. Louis, MO, USA) in 10% ethanol. Images were acquired using the G:BOX Chemi XRQ (Syngene, Cambridge, UK) and analyzed using ImageJ software (US National Institutes of Health, Bethesda, MD, USA).

### Morphology analysis by immunofluorescence

A total of cells at 1 × 10^4^ per well were seeded in a 24-well plate with coverslips at the bottom. The plate was kept at 37°C in an incubator with 5% CO₂ for 48 hours. Next, treatment with NSC305787 was carried out at concentrations of 1.6 and 2.4 μM for A-431 cells, 3.2 and 4.0 μM for AGS cells, and 4.0 and 4.8 μM for HGC-27 cells. After 24h of treatment, the culture medium was removed, the wells were washed 2 times with PBS and the cells were fixed with 3.7% formaldehyde for 15 minutes, permeabilized with 0.5% triton for 10 minutes, and incubated with phalloidin (1:400, Thermo Fisher Scientific, San Jose, CA, USA) for 1h. Then, a new wash with PBS was performed and the slides were prepared with a mounting medium for fluorescence (ProLongTM Gold antifade reagent with DAPI; Thermo Fisher Scientific). Images were performed using fluorescence microscopy (LionHeart FX automated microscope, Biotek, Winooski, VT, USA).

### Cell death analysis by flow cytometry

A total of 1 × 10^5^ cells per well were seeded in 12-well plates in the presence of either the vehicle or NSC305787 (1.6, 2.4, and 4.8 μM for A-431 cells, 3.2, 4, and 8 μM for AGS cells, and 4, 4.8 and 9.6 μM for HGC-27 cells) for 24h. Subsequently, the cells were washed twice with ice-cold PBS and resuspended in a binding buffer containing 1 μg/mL Propidium Iodide (PI) and 1 μg/mL FITC-labeled annexin V (BD Biosciences, San Jose, CA, USA). All samples were acquired by flow cytometry (FACSCalibur; Becton Dickinson, Franklin Lakes, NJ, USA) after 15-minute incubation at room temperature in a light-protected area and analyzed using FlowJo software (Treestar, Inc., San Carlos, CA, USA).

### Western blot analysis

A-431, AGS, and HGC-27 cells were exposed to vehicle or NSC305787 for 24h. Total protein was extracted using a buffer containing 100 mM Tris (pH 7.6), 1% Triton X-100, 2 mM PMSF, 10 mM Na_3_VO_4_, 100 mM NaF, 10 mM Na_4_P_2_O_7_, and 10 mM EDTA. Equal amounts of total protein were separated by SDS-PAGE and identified by western blotting with the relevant antibodies listed below. Protein signals were detected using the SuperSignal^TM^ West Dura Extended Duration Substrate System (Thermo Fisher Scientific) and the G:BOX Chemi XX6 gel doc system (Syngene). Antibodies against PARP1 (#9542), γH2AX (#9718), and α-tubulin (#2144) were purchased from Cell Signaling Technology (Danvers, MA, USA).

### Quantitative PCR (qPCR)

A-431, AGS, and HGC-27 were exposed to vehicle or NSC305787 for 24h. Total RNA was obtained using TRIzol reagent (Thermo Fisher Scientific). The cDNA was synthesized from 1 µg of RNA using a High-Capacity cDNA Reverse Transcription Kit (Thermo Fisher Scientific). Quantitative PCR (qPCR) was performed using a QuantStudio 3 Real-Time PCR System (Thermo Fisher Scientific) and a SybrGreen System and specific primers (Supplementary Table 1), using *HPRT1* and *ACTB* as reference genes. Relative quantification values were calculated using the 2^-ΔΔCT^ equation.^11^ A negative ‘No Template Control’ was included for each primer pair.

### Statistical analysis

Statistical analyses were performed using GraphPad Prism 8 (GraphPad Software, Inc.), SAS System for Windows 9.2 (SAS Institute, Inc., NC, USA), or Stata software (Stata Corp., College Station, TX, USA). The Cox proportional hazard model for regression analysis or a log-rank test (Mantel-Cox) was used to estimate OS, DFS, and DSS. OS was defined as the time from sample collection to the date of death; living patients were censored on the date of the last assessment. DFS was defined as the time interval between treatment and the date of disease progression, relapse, treatment failure, or death; patients alive and without disease progression were censored on the date of the last assessment. DSS was defined as the time from diagnosis or initiation of treatment to the time of death, specifically from the disease. For comparisons, the Student *t*-test or ANOVA and the Bonferroni post-test were used. A p-value <0.05 was considered statistically significant.

## Results

### The pan-cancer analysis identified cancers with high EZR expression

**Fig. 1:**
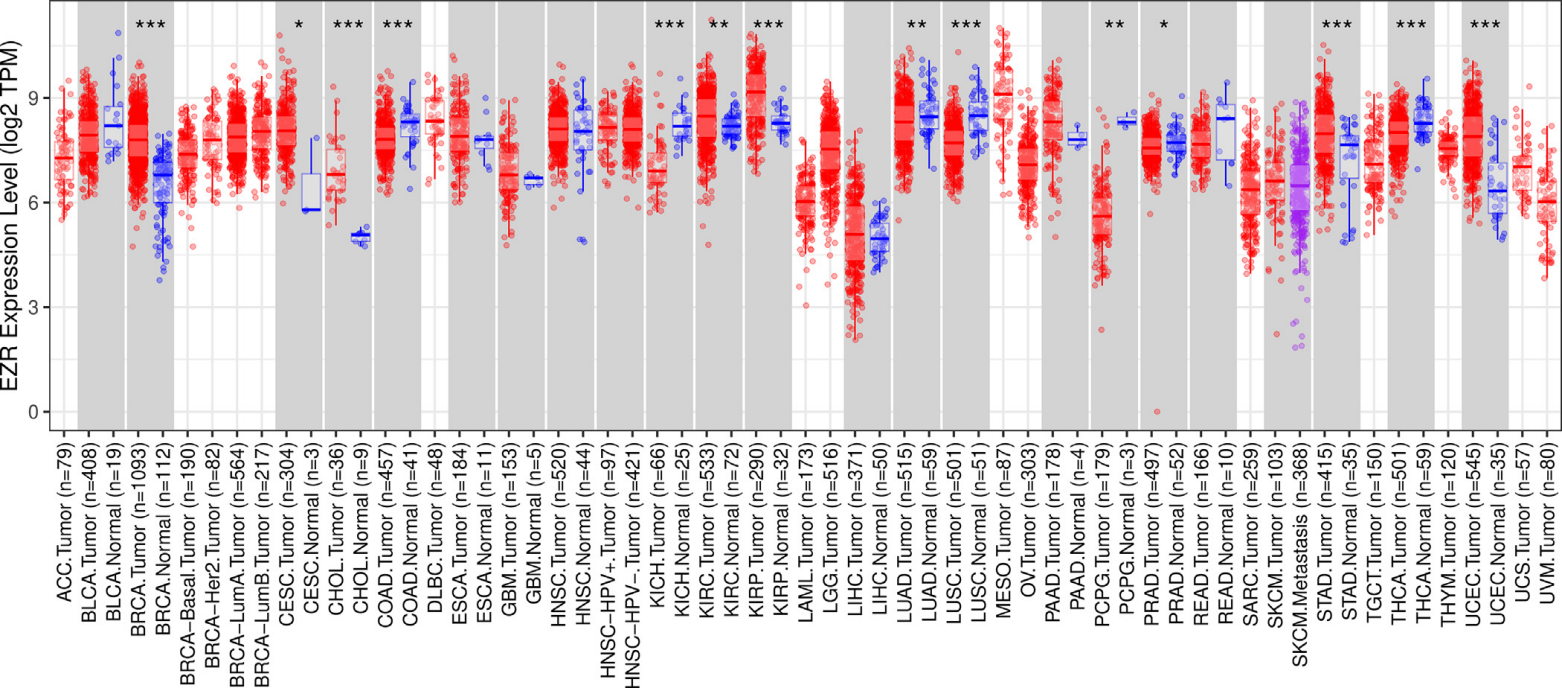
***EZR* expression in a pan-cancer analysis from The Cancer Genome Atlas (TCGA) cohorts.** Data from TCGA cohorts that included 32 types of cancer, 21 of which had data from adjacent normal tissue, were obtained from the **TIMER2**.0 database (http://timer.cistrome.org/). Statistical significance was calculated using the Wilcoxon test; *p < 0.05, **p < 0.01, ***p < 0.001. Abbreviations: ACC, adrenocortical carcinoma; BLCA, Bladder Urothelial Carcinoma; BRCA, Breast Invasive Carcinoma; CESC, Cervical Squamous Cell Carcinoma and Endocervical Adenocarcinoma; CHOL, Cholangiocarcinoma; COAD, Colon Adenocarcinoma; DLBC, Diffuse Large B-Cell lymphoma; ESCA, Esophageal Carcinoma; GBM, Glioblastoma Multiforme; HNSC, Head and Neck Squamous Cell Carcinoma; KICH, Kidney Chromophobe; KIRC, Kidney Renal Clear Cell Carcinoma; KIRP, Kidney Renal Papillary Cell Carcinoma; LAML, Acute Myeloid Leukemia; LGG, Brain Lower Grade Glioma; LIHC, Liver Hepatocellular Carcinoma; LUAD, Lung Adenocarcinoma; LUSC, Lung Squamous Cell Carcinoma; MESO, Mesothelioma; OV, Ovarian Serous Cystadenocarcinoma; PAAD, Pancreatic Adenocarcinoma; PCPG, Pheochromocytoma and Paraganglioma; PRAD, Prostate Adenocarcinoma; READ, Rectum Adenocarcinoma; SARC, Sarcoma; SKCM, Skin Cutaneous Melanoma; STAD, Stomach Adenocarcinoma; TGCT, Testicular Germ Cell Tumors; THCA, Thyroid Carcinoma; THYM, Thymoma; UCEC, Uterine Corpus Endometrial Carcinoma; UCS, Uterine Carcinosarcoma; UVM, Uveal Melanoma.

To begin the exploratory analysis, the authors took advantage of public databases to investigate the transcript levels of *EZR* in different cohorts of TCGA cancer patients. A significant increase in *EZR* expression was observed in patients with breast invasive carcinoma, cervical and endocervical cancer, cholangiocarcinoma, kidney renal clear cell carcinoma, kidney renal papillary cell carcinoma, stomach adenocarcinoma, and uterine carcinosarcoma (all p < 0.05). On the other hand, samples from patients with colon adenocarcinoma, kidney chromophobe, lung adenocarcinoma, lung squamous cell carcinoma, pheochromocytoma and paraganglioma, prostate adenocarcinoma, and thyroid carcinoma showed reduced levels of *EZR* when compared to non-tumor tissues (all p < 0.05; Fig. 1). The role of *EZR* as an oncogene has been widely reported in the literature for several types of tumors, but there are few studies on its potential as a pharmacological target.^3^ Thus, due to limited data on *EZR* in cervical cancer and stomach adenocarcinoma, the authors decided to further the clinical and biological characterization of the role of *EZR* in these cohorts, as well as to investigate the potential of a pharmacological ezrin inhibitor in cellular models of these tumor types.

### EZR expression is associated with relevant biological and molecular processes in cervical squamous carcinoma and stomach adenocarcinoma

**Fig. 2:**
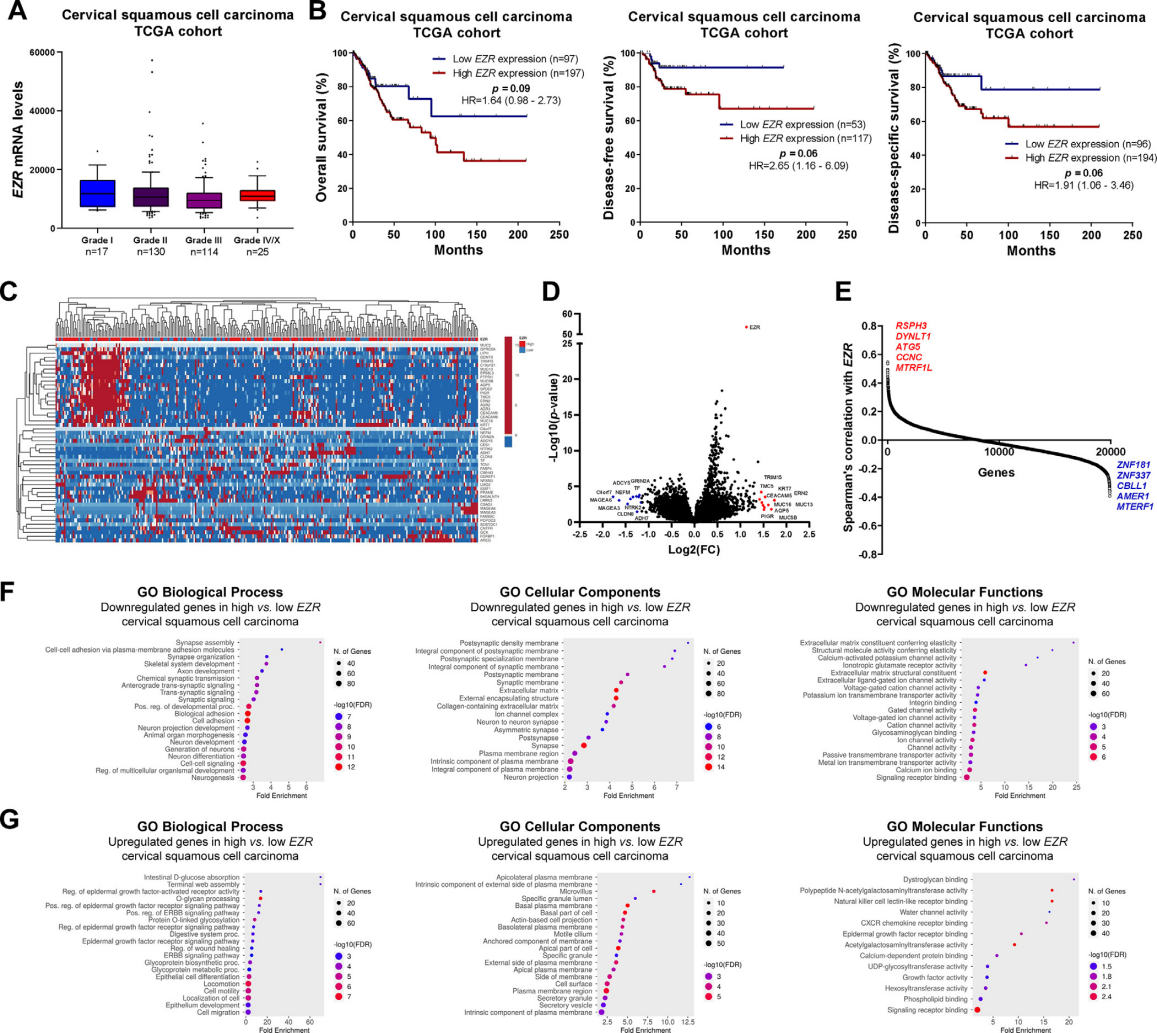
**Association of *EZR* expression and clinical, prognosis, biological and molecular characteristics in cervical squamous cell carcinoma TCGA cohort.** (A) Box plot displaying *EZR* expression distribution between the different grades of cervical squamous cell carcinoma patients. (B) Survival curves were estimated using the Kaplan-Meier method. The Hazard Ratio (HR), 95% Confidence Interval, and p-values are indicated for overall survival, disease-free survival, and disease-specific survival. (C) Heatmap constructed using ClusterVis (https://biit.cs.ut.ee/clustvis/) displaying the top 25 upregulated and 25 downregulated DEGs across high vs. low *EZR* expression. Color intensity represents the z-score within each row. (D) Volcano plot depicting the extent (x-axis) and significance (y-axis) of DEGs, comparing high vs. low *EZR* expression. (E) Graph displaying Spearman's correlation of *EZR* expression with other transcripts’ expression. Representative ShinyGO plots reflect the main 20 biological processes, cellular components, and molecular functions associated with downregulated genes (F) or upregulated genes (G) in high vs. low *EZR* cervical squamous cell carcinoma patients.

**Fig. 3:**
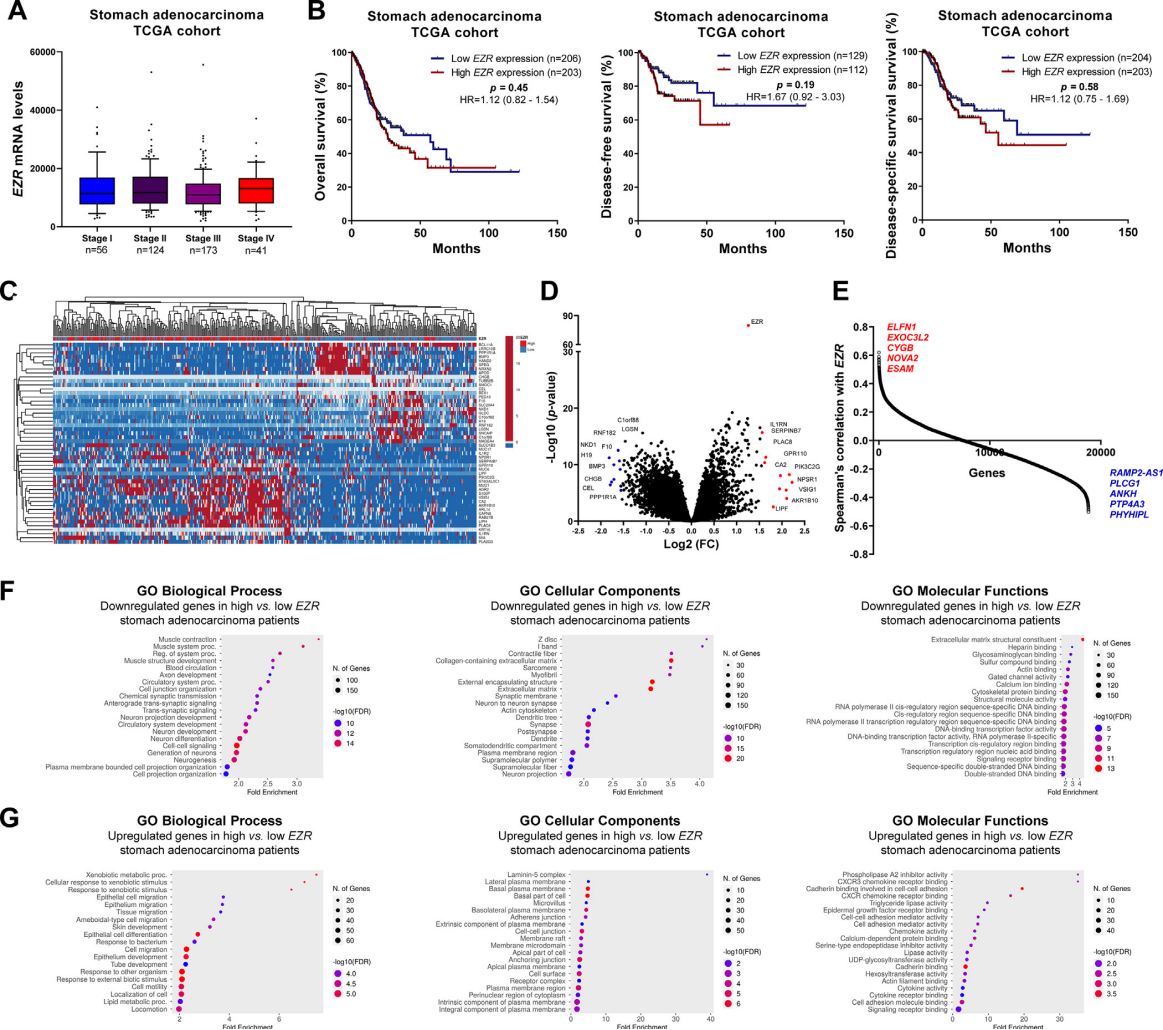
**Clinical, biological, and molecular significance of *EZR* expression in stomach adenocarcinoma TCGA cohort.** (A) Box plot displaying *EZR* expression distribution between the different grades of stomach adenocarcinoma patients. (B) Survival curves were estimated using the Kaplan-Meier method. The Hazard Ratio (HR), 95% Confidence Interval, and p-values are indicated for overall survival, disease-free survival, and disease-specific survival. (C) Heatmap constructed using ClusterVis (https://biit.cs.ut.ee/clustvis/) displaying the top 25 upregulated and 25 downregulated DEGs across high vs low *EZR* expression. Color intensity represents the z-score within each row. (D) Volcano plot depicting the extent (x-axis) and significance (y-axis) of DEGs, comparing high vs. low *EZR* expression. (E) Graph displaying Spearman's correlation of *EZR* expression with other transcripts’ expression. Representative ShinyGO plots reflect the main 20 biological processes, cellular components, and molecular functions associated with downregulated genes (F) or upregulated genes (G) in high vs. low *EZR* stomach adenocarcinoma patients.

In patients with cervical squamous cell carcinoma, *EZR* levels are not associated with disease staging (Fig. 2A), but there is a tendency for increased *EZR* expression to negatively impact disease-free survival (Hazard Ratio [HR = 2.65], 95% Confidence Interval [95 %CI 1.16‒6.09], p = 0.06) and disease-specific survival (HR = 1.91; 95% CI 1.06‒3.46, p = 0.06; Fig. 2B). Functional transcriptomic analysis identified differences in expression between patients with high and low expression of *EZR* (Fig. 2C‒D), as well as the correlation of *EZR* with several other genes (Fig. 2E). Those differently expressed genes were associated with the downregulation or upregulation of relevant biological processes, cellular components, and molecular functions, of which the authors would like to highlight cell adhesion, cell-cell signaling, development, cell mobility, migration, epithelial differentiation, and the EGFR-mediated signaling pathway (Fig. 2F-G). Similarly, *EZR* expression was not associated with staging (Fig. 3A) or survival outcomes (Fig. 3B) in the stomach adenocarcinoma cohort. High and low *EZR* expression allowed the identification of distinct gene expression signatures in patients with stomach adenocarcinoma (Fig. 3C‒E). Among the cellular processes, cellular components, and associated molecular functions, the authors would like to highlight cell-cell signaling, cell motility, migration, development, differentiation, and signaling receptor binding (Fig. 3F‒G). The association of *EZR* expression with clinical and laboratory characteristics in patients with cervical squamous carcinoma and stomach adenocarcinoma are described in Tables 1 and 2.

**Table 1:** Association of *EZR* expression with clinical and molecular factors in TCGA cervical squamous cell carcinoma cohort.

Clinicopathological factors		*EZR*, n (%)		
	n	Low	High	p-value^a^
Total	294	97 (33.0)	197 (67.0)	
Age				0.812
< 60	231	77 (33.3)	154 (67.7)	
≥ 60	63	20 (31.7)	43 (68.3)	
Total	234	77 (32.9)	157 (67.1)	
Primary tumor size (TNM system)				0.736
T1 and T2	206	67 (32.5)	139 (67.5)	
T3 and T4	28	10 (35.7)	18 (64.3)	
Total	186	60 (32.3)	126 (67.7)	
Regional lymph node metastasis (TNM system)				0.136
N0	129	46 (35.7)	83 (64.3)	
N1, N2 and N3	57	14 (24.6)	43 (75.4)	
Total	120	47 (39.2)	73 (60.8)	
Distant metastasis (TNM system)				0.079
M0	111	41 (36.9)	70 (63.1)	
M1	9	6 (66.7)	3 (33.3)	
Total	262	92 (35.1)	170 (64.9)	
Histological grade				0.337
G1	17	5 (29.5)	12 (70.6)	
G2	130	41 (31.5)	89 (68.5)	
G3	115	46 (40)	69 (60)	

a Missing values were excluded from calculation of p-values.

**Table 2:** Association of *EZR* expression with clinical and molecular factors in TCGA stomach adenocarcinoma cohort.

Clinicopathological factors		*EZR*, n (%)		
	n	Low	High	p-value^a^
Total	408	203 (49.8)	205 (50.2)	
Age				**0.05**
< 60	121	69 (57.0)	52 (43.0)	
≥ 60	287	134 (46.7)	153 (53.3)	
Total	412	206 (50.0)	206 (50.0)	
Sex				0.92
Female	145	73 (50.3)	72 (49.7)	
Male	267	133 (49.8)	134 (50.2)	
Total	403	203 (50.4)	200 (49.6)	
Primary tumor size (TNM system)				0.89
T1 and T2	108	55 (54.4)	53 (53.6)	
T3 and T4	295	148 (50.2)	147 (49.8)	
Total	394	198 (50.3)	196 (49.7)	
Regional lymph node metastasis (TNM system)				0.71
N0	122	63 (51.6)	59 (48.4)	
N1, N2 and N3	172	135 (49.6)	137 (50.4)	
Total	392	200 (51.0)	192 (49.0)	0.47
Distant metastasis (TNM system)				
M0	367	189 (51.5)	178 (48.5)	
M1	25	11 (44.0)	14 (56.0)	
Total	394	199 (50.5)	195 (49.5)	
TNM stage				0.70
Stage I and II	180	89 (49.4)	91 (50.6)	
Stage III and IV	214	110 (51.4)	104 (48.6)	
Total	375	185 (49.3)	190 (50.7)	
Molecular subtype				**<0.0001**
Genomically stable	50	36 (72.0)	14 (28.0)	
Epstein-Barr virus	29	10 (34.5)	19 (65.5)	
Chromosomal instability	223	124 (55.6)	99 (44.4)	
Microsatellite instability	73	15 (20.5)	58 (79.5)	
Total	403	202 (50.1)	201(49.9)	
Histological grade				0.48
G1	12	8 (66.7)	4 (33.3)	
G2	144	73 (50.7)	71 (49.3)	
G3	247	121 (49.0)	126 (51.0)	

a Missing values were excluded from calculation of p-values.

### Pharmacological inhibition of ezrin reduces viability and clonal growth in cervical and stomach cancer cells

**Fig. 4:**
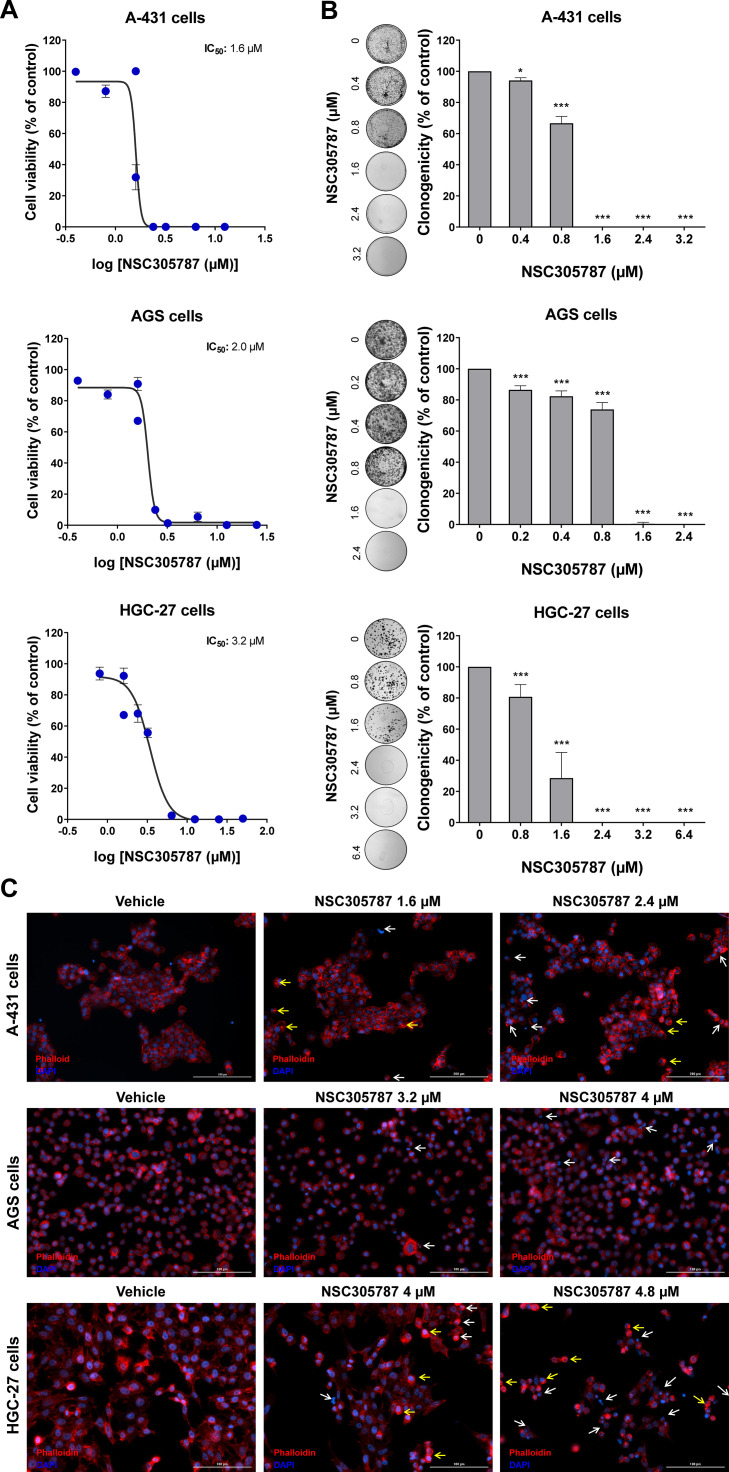
**Pharmacological inhibition of ezrin reduces cell viability and clonal growth of cervical and gastric cancer cells.** (A) Dose-response cytotoxicity was evaluated with the Methylthiazoletetrazolium (MTT) assay. A-431, AGS, and HGC-27 cells were treated with vehicle or different concentrations of NSC305787 for 24h. Values are expressed as the percentage of viable cells for each condition relative to vehicle-treated cells. Results are shown as mean ± SD of at least 3 independent experiments. (B) Colony formation of the cells treated with vehicle or NSC305787 for 10–15 days. The bar graph represents the mean ± SD of the relative number of colonies (% of control). * p < 0.05, *** p < 0.001; ANOVA and Bonferroni post-test. (C) Immunofluorescence analysis of A-431, AGS, and HGC-27 treated with vehicle or NSC305787 for 24h, displaying phalloidin (red) and DAPI (blue) staining. The white arrows indicate pyknotic nuclei and the yellow arrows indicate actin inclusion bodies. Scale bar 100 or 200 µm.

**Fig. 5:**
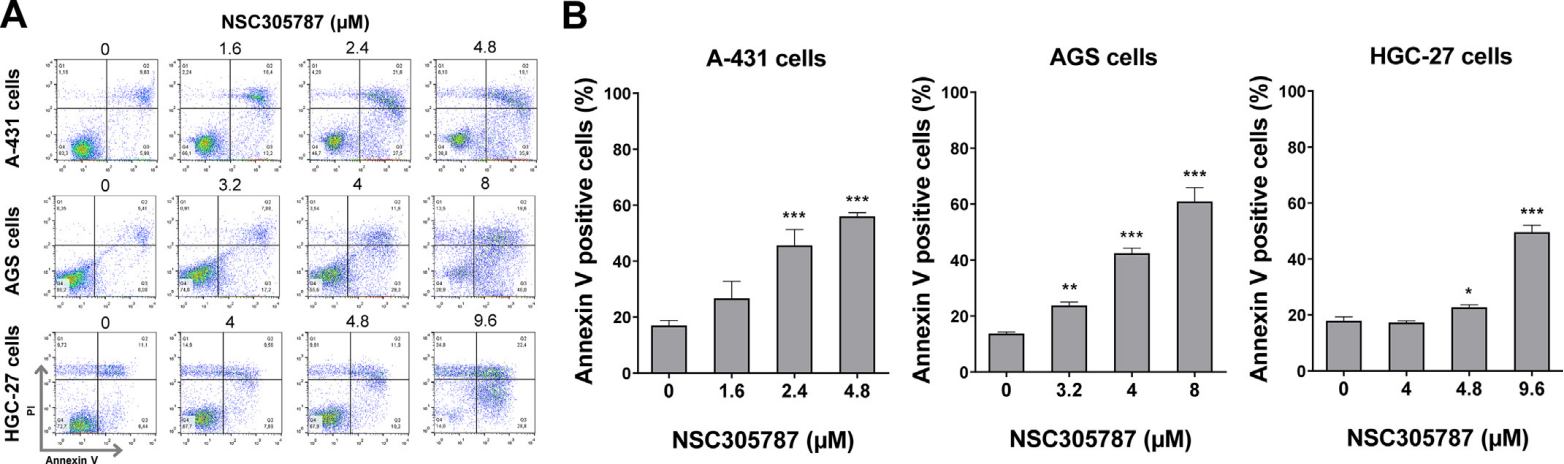
**NSC305787 induces cell death in cervical and gastric cancer cells.** (A) Apoptosis was detected by flow cytometry in A-431, AGS, and HGC-27 cells treated with vehicle or increasing concentrations of NSC305787 for 24 hours using annexin V/Propidium Iodide (PI) staining. Representative dot plots are shown for each condition; the upper and lower right quadrants (Q2 + Q3) cumulatively contain the cell death population (annexin V^+^ cells). (B) The bar graphs represent the mean ± SD from at least three independent experiments quantifying cell death. The p-values and cell lines are indicated in the graphs; *p < 0.05, **p < 0.01, ***p < 0.0001; ANOVA test and Bonferroni post-test.

Next, the potential antineoplastic effects of the pharmacological ezrin inhibitor NSC305787 were investigated in cervical (A-431) and gastric (AGS and HGC-27) cancer cells. NSC305787 dose-dependently reduced the viability of all tested cell models, with IC50 values of 1.6, 2.2, and 3.2 µM for A-431, AGS, and HGC-27 cells, respectively (Fig. 4A). Notably, long-term exposure strongly reduced colony formation at concentrations starting at 1.6 µM for A-431 and AGS cells and 2.4 µM for HGC-27 cells (all p < 0.05; Fig. 4B). Morphological analysis indicates the presence of cells with pyknotic or fragmented nuclei, in addition to the presence of actin inclusion bodies, suggesting cytoskeletal disorganization and apoptosis (Fig. 4C). Flow cytometry analyses indicate that exposure to NSC305787 induces cell death in a concentration-dependent manner in A-431, AGS, and HCG-27 cells (Fig. 5).

### Pharmacological inhibition of ezrin induces molecular markers of cell death

**Fig. 6:**
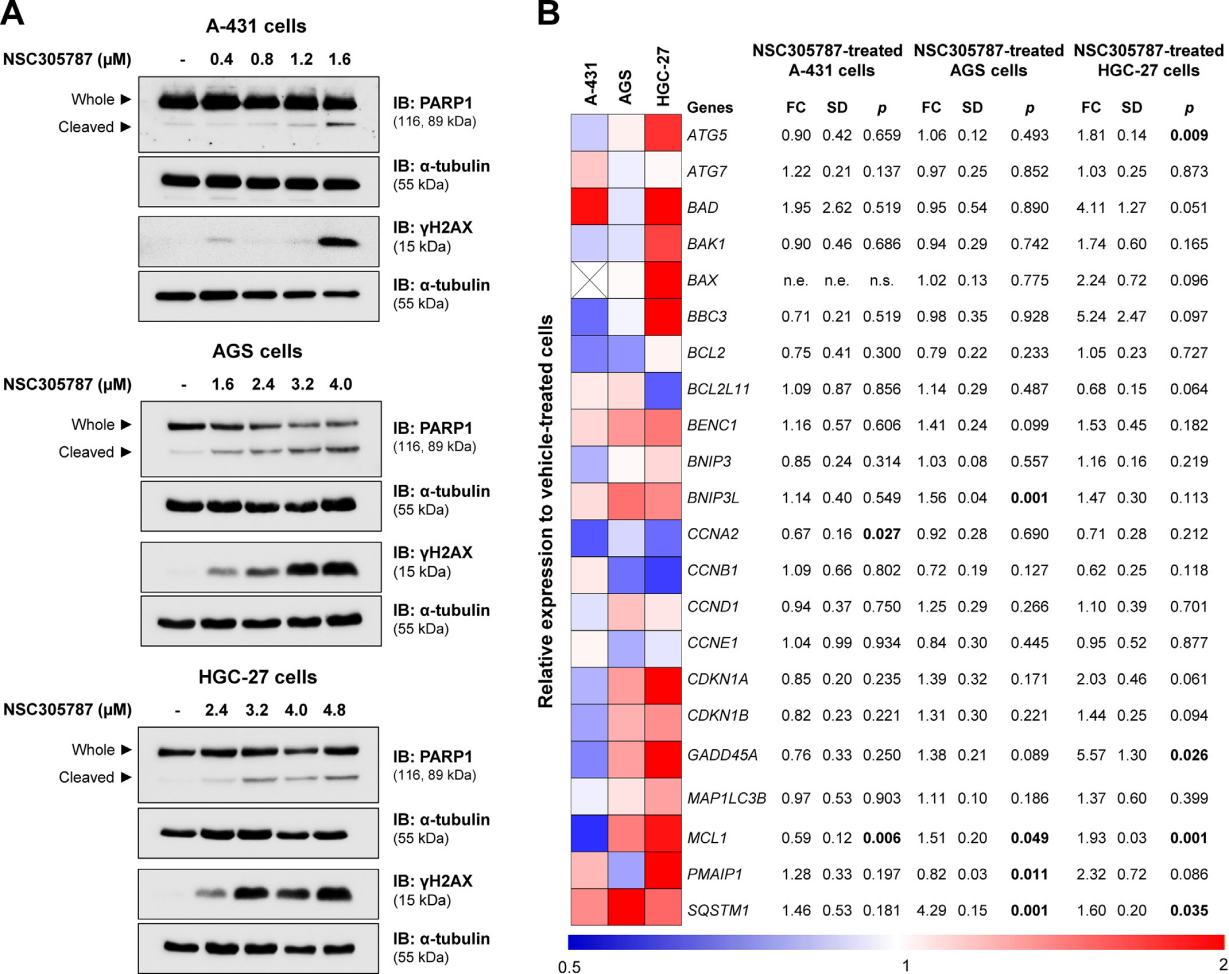
**NSC305787 induces apoptosis and DNA damage markers and modulates survival-related genes in cervical and gastric cancer cells.** (A) Western blot analysis PARP1 and γH2AX in total cell extracts from A-431, AGS, and HGC-27 cells treated with vehicle or NSC305787 for 24h. The membranes were incubated with the indicated antibodies and developed with the SuperSignal™ West Dura Extended Duration Substrate and Gel Doc XR + system. (B) Heatmap for the gene expression analysis in A-431, AGS, and HGC-27 cells treated with vehicle or NSC305787 for 24h. The data represent the fold change of vehicle-treated cells, and downregulated and upregulated genes are shown in blue and red, respectively. Fold-Change (FC), Standard Deviation (SD), and p-values are indicated, Student *t*-test.

From a molecular point of view, NSC305787 induces apoptosis markers (cleaved PARP1) and DNA damage (γH2AX) in the evaluated cervical and gastric cancer cell models (Fig. 6A). To provide new insights into ezrin inhibitor action in the studied models, a panel of genes involved in cell cycle progression, apoptosis, DNA damage, and autophagy was investigated. In A-431 cells, drug treatment reduced *MCL1* and *CCNE1* expression (p < 0.05). In AGS cells, an increase in *BNIP3L, MCL1, PMAIP1,* and *SQSTM1* upon exposure to NSC305787 was observed (p < 0.05). The expression levels of *ATG5, GADD45A, MCL1,* and *SQSTM1* were upregulated in HGC-27 cells treated with the pharmacological inhibitor of ezrin (p < 0.05; Fig. 6B).

## Discussion

Increasing attention has been given to the functional characterization of the ezrin protein and its relationship with different types of cancer. Studies have highlighted its role in important cellular processes and the correlation between high levels of *EZR* expression and tumor progression and metastasis, as well as worse prognosis.^3^^,^^12^ In cervical carcinoma, the ezrin protein is highly expressed in all cervical cancer cell lines and tissues compared with normal cervical tissues and predicts poor prognosis in cervical cancer patients.^13^^,^^14^ *EZR* expression is also associated with the severity of HPV-associated squamous intraepithelial lesions.^15^ *EZR* silencing reduces migration, invasion, and mesenchymal markers expression, and PI3K/AKT activation in HeLa and SiHa cells.^13^^,^^14^ Similarly, *EZR* was found to be highly expressed in gastric cancer compared to non-tumor mucosae, which is associated with age, tumor size, location, differentiation stage, depth of invasion, vessel invasion, lymph node, and distant metastasis, and it is an independent prognostic factor in patients with gastric carcinoma.^16^^,^^17^ Furthermore, increased *EZR* expression is related to *Helicobacter pylori* infection, a risk factor for gastric cancer.^18^ The present findings corroborate these previous studies, indicating that the high expression of *EZR* may contribute to the malignant phenotype of these neoplasms.

Among the main biological processes and molecular functions, the authors highlight that a higher expression of *EZR*, both in cervical carcinoma and gastric adenocarcinoma, is related to the reduction of biological processes associated with signaling and cell-cell contact, negatively regulating cell junction, a factor that can facilitate the development of metastasis. Among the biological processes with increased function in patients with high *EZR* expression, there is cell motility, locomotion, migration, and epithelial differentiation, all factors strongly related to tumor formation. The increased molecular functions, on the other hand, highlight a strong association with growth factors, also related to tumor development and association with the binding of actin filaments, a function of ezrin already well described in the literature.^3^^,^^19^

Given the above, *EZR* proved to be a potential therapeutic target and inhibitor molecules began to be tested in preclinical studies for various tumors.^3^^,^^19^ Two pharmacological ezrin inhibitors have been better characterized in cancer models, NSC305787 and NSC668394, with NSC305787 being considered more potent and with a more favorable pharmacokinetic profile,^6^^,^^7^ which led us to choose this compound for the studies *in vitro* in models of cervical and gastric cancer. NSC305787 reduced cell viability, and colony formation, and led to morphological changes compatible with apoptosis. From a molecular point of view, drug treatment induced markers of DNA damage and apoptosis. These findings are in line with those previously described in pancreatic cancer and acute leukemia.^20^^,^^21^

In exploratory analysis of gene expression, pharmacological inhibition reduced *MCL1* and *CCNA2* expression in A-431 cells. MCL1 is a BCL2 family protein that can act in the control of apoptosis, interfering at an early stage in a cascade of events that lead to the release of cytochrome c from mitochondria.^22^ The relationship between MCL1 and cancer is well described in the literature, studies have shown that its overexpression contributes to cell survival and resistance to various chemotherapeutic agents.^23^^,^^24^ Research has shown that there is overexpression of MCL1 in cervical cancer, while adjacent normal tissue showed slight or no expression of this protein. Furthermore, increased levels of MCL1 are closely associated with the pathogenesis of cervical cancer, being associated with histological grade, tumor size, and lymph node involvement, and positively correlated with poor prognosis.^25^ In contrast, the MCL1 gene was significantly more expressed in both gastric cancer cell lines, which could act as a drug resistance mechanism.^26^ Cyclin A2 is associated with both CDK1 and CDK2 and has roles in both S phase and mitosis.^27^ Consistent with its role as a key cell cycle regulator, expression of this protein is elevated in a variety of tumors.^28^^,^^29^ In addition to potentially disrupting the cell cycle and checkpoints directly, cyclin A may also contribute to tumor formation through the phosphorylation of other oncogenic and tumor suppressor proteins.^27^ Research has shown that CCNA2 is significantly increased in cervical cancer tissues, suggesting a possible mechanism of anomalous mitosis in this condition.^30^ The increase in this expression reflects a high rate of cell proliferation once the tumor has been developed.^31^

In the present study, gastric cancer cells showed increased expression of genes involved in the autophagy process, such as ATG5 and SQSTM1, after exposure to NSC305787. *ATG5* encodes an E1-type activating enzyme essential for autophagy and for the transport of molecules from the cytoplasm to the vacuole,^32^ while *SQSTM1* encodes the p62 protein, which acts as a receptor for the degradation of ubiquitinated proteins by autophagic or proteasome pathways.^33^ These findings indicate that *EZR* inhibition in gastric cancer cell lines may activate genes related to autophagy, which deserves further investigation. Furthermore, in the AGS cells, there was an increase in the expression of *BNIP3L*, which was initially recognized as coding for a pro-apoptotic protein of the BCL2 family. Specifically, BNIP3L interacts with the pro-apoptotic proteins of the BAX and BAK family to increase the permeability of the outer mitochondrial membrane. However, the most important activity of BNIP3L in the cell has been found to be the promotion of mitochondrial autophagy (mitophagy) through the recruitment of autophagosomes in response to cellular or environmental stress.^34^^,^^35^ Also in this cell line, there was a decrease in the expression of the *PMAIP1*, which belongs to the pro-apoptotic subfamily within the BCL2 protein family and promotes the activation of caspases, changes in the mitochondrial membrane and efflux of apoptogenic proteins from the mitochondria.^36^^,^^37^

Additionally, in HGC-27 cells, there was an increase in the expression of *BAD* and *GADD45A. BAD* encodes a protein member of the BCL2 family, which are regulators of programmed cell death. This protein positively regulates cell apoptosis by forming heterodimers with *BCL2L1* and *BCL2*, reversing its death repressor activity.^38^ *GADD45A* is a member of a group of genes whose transcription levels are increased after stressful conditions of growth arrest and DNA damage.^39^^,^^40^

In summary, this study reveals elevated EZR expression in cervical and gastric carcinoma, highlighting its potential as a molecular target for treating these cancers. The present preclinical findings pave the way for further research and support the need for clinical trials to explore the effectiveness of pharmacological EZR inhibitors in patients with cervical and gastric carcinoma.

## CRediT authorship contribution statement

**Maria Fernanda Lopes Carvalho:** Conceptualization, Methodology, Validation, Formal analysis, Investigation, Data curation, Writing – original draft, Visualization. **Carolina Santana Calicchio:** Conceptualization, Methodology, Validation, Formal analysis, Investigation, Data curation, Writing – original draft, Visualization. **Bruna Oliveira de Almeida:** Methodology, Validation, Data curation, Writing – review & editing. **Livia Bassani Lins de Miranda:** Methodology, Validation, Data curation, Writing – review & editing. **Jean Carlos Lipreri da Silva:** Methodology, Validation, Data curation, Writing – review & editing. **Keli Lima:** Conceptualization, Methodology, Validation, Investigation, Data curation, Writing – review & editing. **João Agostinho Machado-Neto:** Conceptualization, Methodology, Validation, Formal analysis, Resources, Writing – original draft, Visualization, Supervision, Project administration, Funding acquisition.

## Conflicts of interest

The authors declare no conflicts of interest.

## Appendix Supplementary materials

Image, application 1
