# Supplementary material for: Transcriptomics analysis identified ezrin as a potential druggable target in cervical and gastric cancer cells
Source: Clinics (Sao Paulo). 2024 Jul 6;79:100422. doi: 10.1016/j.clinsp.2024.100422 (PMC11276928; doi:10.1016/j.clinsp.2024.100422)
Supplement: Supplementary file 1 [file mmc1.doc]

**CLINICS-D-24-00105_Supplementary Material**

**Supplementary Table 1 Sequences and concentrations of primers used in the quantitative PCR reaction and cellular processes related to the analyzed genes of interest.**

| **Gene** | **Sequence** | **Concentration** | **Cellular process** |
| --- | --- | --- | --- |
| *ATG5* | FW: GGGCCATCAATCGGAAAC | 300 nM | Autophagy |
|  | RV: AGCCACAGGACGAAACAG |  |  |
| *ATG7* | FW: CGTTGCCCACAGCATCATCTTC | 300nM | Autophagy |
|  | RV: TCCCATGCCTCCTTTCTGGTTC |  |  |
| *BAD* | FW: CACCAGCAGGAGCAGCCAAC | 300 nM | Apoptosis |
|  | RV: CGACTCCGGATCTCCACAGC |  |  |
| *BAK1* | FW: TGACTTCACCAAGATTGCCA | 300 nM | Apoptosis |
|  | RV: AGTCAGGCCATGCTGGTAGAC |  |  |
| *BAX* | FW: GAGCTGCAGAGGATGATTGC | 300 nM | Apoptosis |
|  | RV: CAGCTGCCACTCGGAAAA |  |  |
| *BBC3* | FW: GACCTCAACGCACAGTACGAG | 300 nM | DNA damage/ Apoptosis |
|  | RV: AGGAGTCCCATGATGAGATTG |  |  |
| *BCL2* | FW: ATGTGTGTGGAGAGCGTCAA | 300nM | Apoptosis |
|  | RV: ACAGTTCCACAAAGGCATCC |  |  |
| *BCL2L11* | FW: ATGTCTGACTCTGACTCTCG | 300 nM | Apoptosis |
|  | RV: CCTTGTGGCTCTGTCTGTAG |  |  |
| *BECN1* | FW: ACAAATCTAAGGAGCTGCCGTTA | 300 nM | Autophagy |
|  | RV: TGCACACAGTCCAGGAAAGC |  |  |
| *BNIP3* | FW: ATATGGGATTGGTCAAGTCGG | 300 nM | Apoptosis/ Autophagy |
|  | RV: CGCTCGTCCTCATGCT |  |  |
| *BNIP3L* | FW: ACACCAGCAGGGACCATAGC | 300 nM | Apoptosis/ Autophagy |
|  | RV: TTTCTTCAAAGCTCGACTTCC |  |  |
| *CCNA2* | FW: GCCTTTCATTTAGCACTCTACA | 300 nM | Cell cycle |
|  | RV: CAGGGTATATCCAGTCTTTCG |  |  |
| *CCNB1* | FW: GTCTCCATTATTGATCGGTTCATG | 300 nM | Cell cycle |
|  | RV: CCAATTTCTGGAGGGTACATTTCT |  |  |
| *CCND1* | FW: CTCGGTGTCCTACTTCAAATG | 300 nM | Cell cycle |
|  | RV: AGCGGTCCAGGTAGTTCAT |  |  |
| *CCNE1* | FW: TATATGGCGACACAAGAAAATG | 300 nM | Cell cycle |
|  | RV: GTGCAACTTTGGAGGATAGA |  |  |
| *CDKN1A* | FW: TGTCACTGTCTTGTACCCTTGT | 300 nM | Cell cycle |
|  | RV: GCCGGCGTTTGGAGTGGTAG |  |  |
| *CDKN1B* | FW: ACTCTGAGGACACGCATTTGGT | 300 nM | Cell cycle |
|  | RV: TCTGTTCTGTTGGCTCTTTTGTT |  |  |
| *GADD45A* | FW: AAGGATGGATAAGGTGGGG | 300 nM | DNA damage |
|  | RV: CTGGATCAGGGTGAAGTGG |  |  |
| *MAP1LC3B* | FW: AAGGCGCTTACAGCTCAATG | 300 nM | Autophagy |
|  | RV: CTGGGAGGCATAGACCATGT |  |  |
| *MCL1* | FW: GTAATAACACCAGTACGGACGG | 300 nM | Apoptosis |
|  | RV: TCCCGAAGGTACCGAGAGAT |  |  |
| *PMAIP1* | FW: CGCGCAAGAACGCTCAACC | 300 nM | DNA damage/  Apoptosis |
|  | RV: CACACTCGACTTCCAGCTCTGCT |  |  |
| *SQSTM1* | FW: TGAGGAACAGATGGAGTCGGATAA | 300 nM | Autophagy |
|  | RV: GGGACTGGAGTTCACCTGTAGACG |  |  |
| *HPRT1* | FW: GAACGTCTTGCTCGAGATGTGA | 150 nM | Reference gene |
|  | RV: TCCAGCAGGTCAGCAAAGAAT |  |  |
| *ACTB* | FW: AGGCCAACCGCGAGAAG | 150 nM | Reference gene |
|  | RV: ACAGCCTGGATAGCAACGTACA |  |  |

FW, Forward; RV, Reverse. Gene names conform to the HUGO nomenclature for genes and proteins.
